# Iron-dependent ferroptosis in cardiac microvascular endothelial cells: a key link between dysregulated iron homeostasis and microcirculatory injury during myocardial ischemia-reperfusion

**DOI:** 10.3389/fcvm.2026.1761986

**Published:** 2026-05-07

**Authors:** Xiaoya Li, Qingbo Shi, Zhiqiang Wang, Zhiwen Zhang, Muwei Li

**Affiliations:** 1 Health Science Center, Yangtze University, Jingzhou, China; 2 Department of Cardiology, Fuwai Central China Cardiovascular Hospital, Central China Fuwai Hospital of Zhengzhou University, Zhengzhou, China

**Keywords:** ferritinophagy, ferroptosis, iron homeostasis, microcirculatory injury, myocardial ischemia-reperfusion

## Abstract

Despite successful epicardial recanalization, effective tissue reperfusion frequently remains limited by cardiac microvascular dysfunction, including endothelial swelling, barrier leakage, leukocyte-platelet adhesion, and microvascular obstruction/no-reflow. A growing body of work links these microcirculatory phenotypes to iron-driven phospholipid peroxidation and ferroptosis. During ischemia-reperfusion, dysregulated iron handling expands the labile iron pool through iron import, heme/iron release, and ferritinophagy, while antioxidant defenses become compromised. Cardiac microvascular endothelial cells (CMECs) appear particularly vulnerable to this iron-dependent lipid peroxidation cascade, and ferroptotic or sublethally injured CMECs can propagate microvascular injury by weakening junctional integrity, increasing edema, and exacerbating perfusion heterogeneity that ultimately amplifies cardiomyocyte hypoxia and death. In this review, we summarize the mechanistic basis of endothelial ferroptosis under iron dyshomeostasis, propose an integrated CMEC-centered framework connecting iron dysregulation to microcirculatory injury during reperfusion, and discuss emerging therapeutic opportunities, such as iron chelation, radical-trapping antioxidants, reinforcement of cystine import/antioxidant systems, and microvascular barrier-stabilizing strategies aimed at preserving endothelial function and improving microvascular reperfusion.

## Introduction

Myocardial ischemia-reperfusion (I/R) injury refers to tissue damage that occurs when blood flow is restored to previously ischemic myocardium (1). It most commonly occurs after thrombolysis or primary percutaneous coronary intervention (PCI) for acute myocardial infarction, and also during cardiac surgery, transplantation, and cardiopulmonary bypass (1). Although reperfusion is indispensable for myocardial salvage, abrupt restoration of oxygen and substrates can paradoxically exacerbate injury (1). Early reperfusion triggers a burst of reactive oxygen species (ROS), calcium overload and dysregulation, mitochondrial dysfunction, and sterile inflammation, thereby promoting myocardial stunning, reperfusion arrhythmias, and multiple forms of cell death (1). Yet angiographic success can mask persistent tissue-level hypoperfusion, clinically manifested as microvascular obstruction (MVO) and the coronary no-reflow phenomenon, which are associated with worse outcomes (2, 3).

Because cardiac microvascular endothelial cells (CMECs) line the capillary lumen and regulate capillary patency, barrier integrity, and thrombo-inflammatory interactions, they represent an early and highly susceptible target during reperfusion (4–6). The coronary microcirculation is the final determinant of effective myocardial perfusion (5, 6). CMECs maintain barrier integrity and permeability homeostasis and orchestrate vasomotor tone, leukocyte trafficking, and hemostatic balance (5, 6). Accumulating evidence indicates that microvascular injury actively contributes to adverse remodeling after I/R (2–4). It can induce barrier leakage and edema, facilitate inflammatory cell infiltration, promote capillary plugging and intramyocardial hemorrhage, and ultimately aggravate MVO/no-reflow and impair myocardial repair (2–4). Clinically, preventive strategies remain imperfect and the efficacy of rescue measures after angiographic no-reflow is detected is still uncertain (2, 3). Accordingly, CMEC injury is positioned upstream of endothelial swelling and leakage, leukocyte and platelet plugging, and microthrombus formation that define microvascular obstruction (2–4). Therefore, explaining persistent hypoperfusion after an open artery requires mechanistic insight at the microvascular level (4).

Ferroptosis has emerged as a regulated cell death program implicated in myocardial I/R and may be particularly relevant to the early vulnerability of the cardiac microvascular endothelium during reperfusion (7–10). Importantly, myocardial salvage after reperfusion depends not only on reopening the epicardial artery but also on restoring microvascular patency and endothelial barrier function (2–4). In this review, we summarize current evidence that dysregulated iron homeostasis lowers the ferroptosis threshold in cardiac microvascular endothelial cells (CMECs), and we discuss how endothelial ferroptosis and sublethal lipid peroxidation may act as a mechanistic bridge between iron dyshomeostasis and microcirculatory injury during reperfusion. We further highlight unresolved questions and propose experimental and therapeutic directions to define, detect, and selectively target CMEC ferroptosis to improve microvascular reperfusion and clinical outcomes after myocardial ischemia-reperfusion.

Compared with the rapidly expanding literature on cardiomyocyte ferroptosis, direct evidence and a unified mechanistic framework for CMEC ferroptosis remain limited (7–10). Nonetheless, CMEC-centered studies increasingly link ferroptotic injury to endothelial dysfunction and adverse microvascular outcomes (11–18). In human CMECs exposed to hypoxia/reoxygenation, ferroptosis-associated injury is characterized by increased lipid peroxidation, expansion of the labile iron pool (LIP), mitochondrial structural damage, and impaired migration and tube formation, together with weakened glutathione peroxidase 4 (GPX4)-dependent antioxidant defense (11). Ferroptosis inhibition with ferrostatin-1 partially restores endothelial function, whereas ferroptosis induction with erastin exacerbates these defects (11). In parallel, hypoxia-conditioned CMEC-derived exosomal signaling has been linked to transferrin receptor 1-related ferroptotic regulation in coupled endothelial and cardiomyocyte injury settings (12). *In vivo*, interventions that preserve cystine uptake via solute carrier family 7 member 11 (SLC7A11), also known as xCT, and sustain GPX4-dependent defense have been associated with improved microvascular perfusion and less no-reflow during early reperfusion (13, 14). Endothelial-focused genetic and pharmacological evidence further supports soluble guanylate cyclase-related signaling as a pathway that stabilizes GPX4 during reperfusion and limits no-reflow, with vericiguat showing protection in mouse cardiac I/R models (15). Beyond I/R, pressure overload-driven cardiac hypertrophy and angiotensin II-induced hypertension have also been linked to ferroptosis-related injury in CMECs, with microvascular protection reported when GPX4-linked antioxidant defense is reinforced or upstream regulators are restored (16–18). Collectively, these findings support CMEC ferroptosis as an increasingly testable contributor to microcirculatory dysfunction while underscoring the need for CMEC-targeted *in vivo* validation (2, 4).

In this review, we focus on the proposed axis of iron dyshomeostasis, CMEC ferroptosis, and microcirculatory injury during I/R. We summarize key processes governing endothelial iron uptake, storage, and export, lipid peroxidation and antioxidant defense, and ferritinophagy linked iron mobilization. We also discuss emerging pharmacological strategies aimed at preserving microvascular perfusion and improving functional recovery after I/R (7–10).

## Dysregulation of iron homeostasis and microcirculatory injury

### Iron homeostasis in the cardiac microcirculation

Myocardial oxygen and substrate delivery depends primarily on the coronary microcirculation, a network spanning small arteries and arterioles through capillaries to venules, which ultimately determines oxygen and metabolite delivery to the myocardium (5, 6). Clinical manifestations and outcomes in ischemic heart disease are therefore closely related to the structural and functional integrity of this microvascular bed (5, 6). Cardiac microvascular endothelial cells (CMECs) are among the first cells to experience intense redox shifts and iron/heme-driven stress at the onset of reperfusion in the context of I/R injury, making intracellular iron balance a key determinant of microvascular resilience (4–6). Specifically, iron overload—rather than iron deficiency—increases the labile iron pool (LIP) (19). Excess labile iron catalyzes radical formation, accelerates lipid peroxidation, and lowers the threshold for membrane damage (19).

In the course of ischemia-reperfusion (I/R), reoxygenation and pH restoration lead to mitochondrial stress and thrombo-inflammatory responses. These stresses converge at the microvascular bed, triggering endothelial dysfunction, which manifests as incomplete reperfusion, barrier breach, heterogeneous microvascular circulation, and, in severe cases, the no-reflow phenomenon (4). Disturbed mitochondrial dynamics and dysregulated autophagy/mitophagy further impair stress tolerance and delay recovery (20, 21). Therefore, iron homeostasis plays a key role in determining whether cardiac microvascular endothelial cells (CMECs) can withstand reperfusion stress or progress to persistent microvascular dysfunction (4, 20, 21).

### Ferroptosis and microcirculatory injury

Ferroptosis is an iron-dependent form of regulated cell death driven by oxidative phospholipid damage, typically favored by labile iron expansion and insufficient antioxidant detoxification (19, 22). In CMECs, emerging evidence from hypoxia/reoxygenation (H/R) and myocardial I/R models links ferroptosis-associated lipid peroxidation to impaired migration/tube formation and reduced microvascular perfusion with expansion of no-reflow, and demonstrates protection by ferroptosis-targeted interventions (11, 13–15).

Endothelial-focused evidence is now emerging in cardiac settings. In human CMECs exposed to H/R, ferrostatin-1 mitigates, whereas erastin exacerbates, ferroptosis-associated injury, with concordant changes in endothelial functional endpoints such as wound-healing migration and tube formation, supporting a contributory role of ferroptosis in CMEC dysfunction (11). Importantly, lipid peroxidation can disrupt endothelial junctions early after reperfusion, shifting CMECs toward a leaky, pro-adhesive phenotype that promotes leukocyte/platelet recruitment and microvascular plugging, thereby exacerbating microvascular obstruction and increasing the risk of no-reflow (4). Ferroptosis and inflammation also appear to reinforce each other: oxidized lipids and damage-associated signals can activate adhesion programs and inflammatory cascades, while inflammatory mediators can perturb iron handling and antioxidant systems, further facilitating ferroptosis progression (19, 22).

Collectively, the available cardiac and CMEC studies suggest a consistent pattern during early reperfusion (11, 13–15). Labile iron increases and lipid peroxidation rises, and antioxidant protection becomes insufficient (11, 13–15). This is followed by endothelial dysfunction with weaker junctional stability and reduced reparative capacity, which then promotes leukocyte and platelet accumulation and microvascular plugging (11, 13–15). Across models, ferroptosis-targeted interventions tend to improve endothelial functional measures and are accompanied by better microvascular perfusion and a smaller no-reflow area (11, 13–15).

From a therapeutic perspective, multiple preclinical studies have explored strategies that inhibit ferroptosis or restore GPX4-centered antioxidant capacity in ischemia-reperfusion models (23, 24). Liproxstatin-1 (Lip-1) reduces myocardial injury in mice and restores GPX4 levels (23). Dysregulation of the SLC7A11-glutathione (GSH)-GPX4 axis has also been linked to I/R injury, suggesting that reinforcing this pathway may represent a tractable intervention point (24). Nevertheless, most data remain preclinical and many studies involve both cardiomyocytes and endothelial cells (11, 23, 24). Endothelial-specific and time-resolved causal studies are still needed to define the true contribution and actionable window of cardiac microvascular endothelial ferroptosis within the iron dysregulation to microvascular injury cascade (11, 13–15, 23, 24).

## Mechanisms and biological basis of ferroptosis

### Distinctions between ferroptosis and other forms of cell death

Morphologically, ferroptotic cells commonly show shrunken mitochondria with increased membrane density and reduced or absent cristae, while the nucleus typically lacks the chromatin condensation and fragmentation seen in apoptosis (19, 22, 25–27).

These features align with the current nomenclature of cell death (28). Apoptosis is executed through caspase-dependent signaling and generally proceeds without early membrane rupture, whereas accidental necrosis is characterized by early plasma-membrane disruption and the release of intracellular contents that can amplify inflammation (28). From a mechanistic perspective, ferroptosis is driven by lipid peroxide-mediated membrane damage rather than caspase activation or the pore-forming execution typical of lytic inflammatory death programs (19, 22, 25, 28).

The distinction is especially clear when comparing ferroptosis with pyroptosis (29). Caspase-1 and caspase-4/5/11 are functional inflammatory caspases that induce pyroptosis by cleaving gasdermins and generating membrane pores, which cause cell lysis and inflammatory signaling and are associated with inflammasome (29). Ferroptosis is independent of inflammasome-gasdermin pore formation, although it may play a role in sterile inflammation *in vivo* in case membrane integrity breaks down and damage cues build up, which is influenced by tissue context and timing (19, 28, 29).

Relative to the environment of I/R injury, reperfusion supplies ROS, as well as redox-active iron, which facilitates iron-dependent redox chemistry that promotes lipid peroxidation and enhances vulnerability to ferroptosis (30). In addition to cardiomyocytes, endothelial ferroptosis has been attributed to microvascular dysfunction in response to the post-I/R injury, suggesting that endothelial ferroptosis may represent a mechanistic link between iron dyshomeostasis and microcirculatory injury (13). Anti-ferroptotic interventions and maintenance of SLC7A11-sensitive access to GSH have been suggested to mediate I/R injury, thereby indicating ferroptosis as a therapeutically exploitable pathway in ischemic heart disease (31).

In cardiac microvascular endothelial cells subjected to ischemia-reperfusion-like stress, several regulated cell death programs have been reported alongside ferroptosis, including apoptosis and inflammasome-linked pyroptosis, and microvascular endothelium can also execute necroptosis when exposed to pro-death inflammatory signaling (32–34). For example, H/R-induced CMEC injury has been linked to caspase-dependent apoptotic signaling (32), and inflammasome/NOD-like receptor family pyrin domain containing 3 (NLRP3)-associated inflammatory signaling has also been implicated in CMEC injury under I/R-like stress (33). However, head-to-head quantification of ferroptosis vs. apoptosis/pyroptosis/necroptosis specifically in CMECs within the same I/R model and time window remains unavailable; therefore, the relative contribution of each program cannot be reliably stated (32–34). Yet most studies examine one pathway in isolation, leaving the *in vivo*, quantitative contribution of each death modality largely unresolved (32–34). This gap matters because endothelial loss is likely heterogeneous in both space and time, differing between vascular segments such as capillary and venular beds and evolving from early to late phases of reperfusion (32–36). Moreover, blocking one regulated death route may reveal latent pathways or redirect signaling into alternative modes of death (35, 36). A practical path forward is to pair time-resolved endothelial phenotyping with multiplexed cell-death measurements within the same model, ideally coupled to endothelial lineage tracing (37, 38). For example, lipid peroxidation together with dependence on GPX4 and acyl-CoA synthetase long-chain family member 4 (ACSL4) can support ferroptosis; caspase activation can indicate apoptosis; gasdermin cleavage together with IL-1β release can indicate pyroptosis; and phosphorylation of receptor-interacting protein kinase 1 (RIPK1), receptor-interacting protein kinase 3 (RIPK3), and mixed lineage kinase domain-like pseudokinase (MLKL) can report necroptosis (19, 25, 29, 32–34, 39, 40). Such designs would allow observing the relative contribution of every mode of death in cardiac microvascular endothelium and would help to clarify when ferroptosis plays a leading role or cooperates during myocardial reperfusion injury (32–34, 37, 38).

### Key molecules and signaling pathways

**Figure 1:**
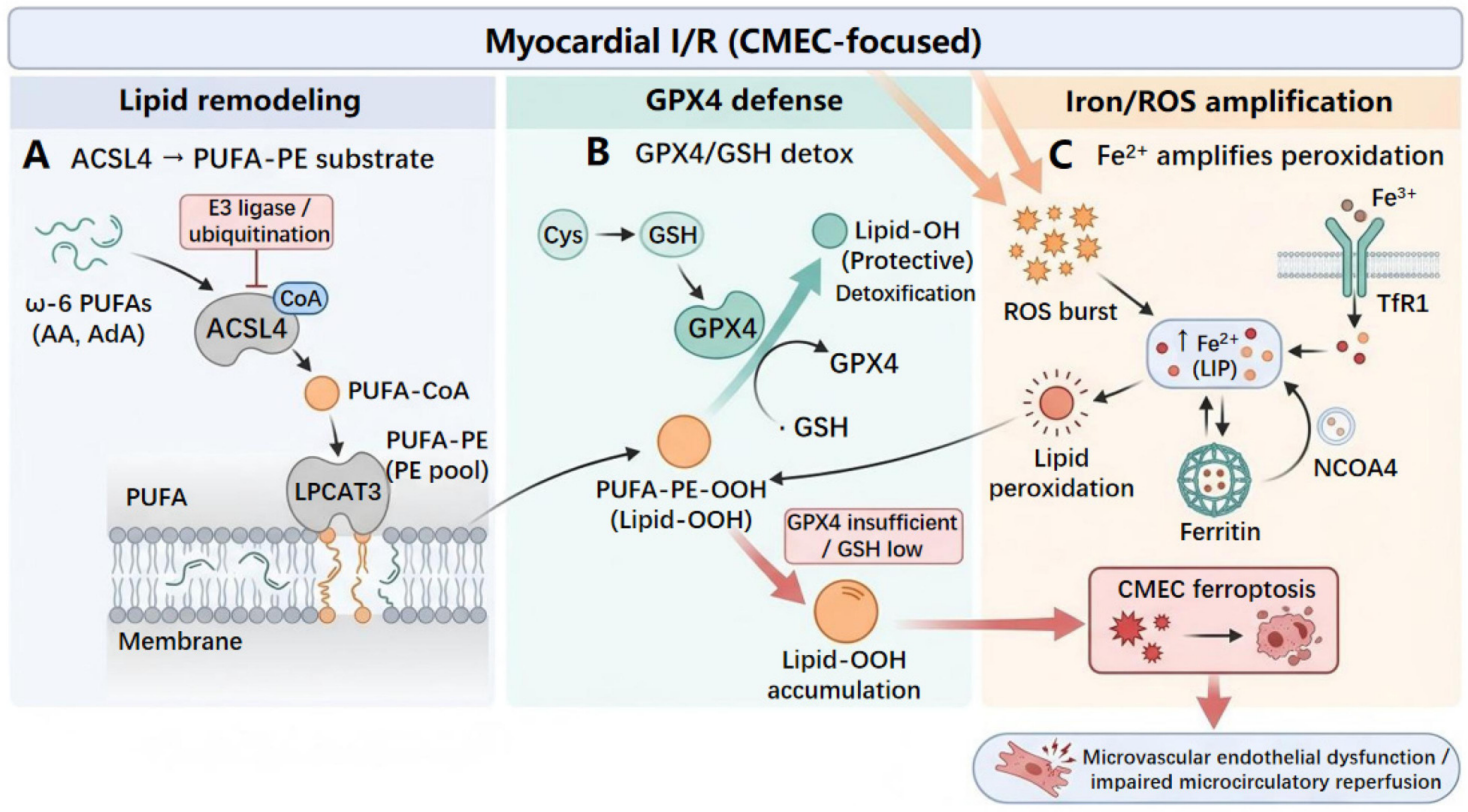
ACSL4-driven PUFA-PE remodeling, GPX4/GSH defense, and iron/ROS amplification converge to trigger ferroptosis during myocardial ischemia-reperfusion. **(A)** ACSL4 preferentially converts *ω*-6 PUFAs (AA, AdA) to PUFA-CoA, which are incorporated by LPCAT3 into phosphatidylethanolamine (PE), enriching membranes with peroxidation-prone PUFA-PE substrates. E3 ligase–mediated ubiquitination restrains ACSL4 and limits PUFA-PE substrate supply. **(B)** GPX4 uses glutathione (GSH) to detoxify phospholipid hydroperoxides (PUFA-PE-OOH/Lipid-OOH) into protective lipid alcohols (Lipid-OH), thereby limiting lipid peroxide accumulation. When GPX4 activity is insufficient or GSH is low, Lipid-OOH accumulates and propagates ferroptotic signaling. **(C)** During I/R, ROS burst and increased labile Fe²⁺ (LIP)—driven by TfR1-mediated iron uptake and ferritin iron storage/release, including NCOA4-mediated ferritin turnover/iron release (ferritinophagy)—amplify lipid peroxidation and Lipid-OOH formation, culminating in CMEC ferroptosis and contributing to microvascular endothelial dysfunction and impaired microcirculatory reperfusion. I/R, ischemia-reperfusion; ACSL4, acyl-CoA synthetase long-chain family member 4; PUFA, polyunsaturated fatty acids; PE, phosphatidylethanolamine; AA, arachidonic acid; AdA, adrenic acid; CoA, coenzyme A; LPCAT3, lysophosphatidylcholine acyltransferase 3; GPX4, glutathione peroxidase 4; GSH, glutathione; Cys, cysteine; ROS, reactive oxygen species; Fe^2^⁺, ferrous iron; Fe³⁺, ferric iron; LIP, labile iron pool; TfR1, transferrin receptor 1; NCOA4, nuclear receptor coactivator 4.

Accumulating evidence suggests that the ferroptosis threshold is determined not only by GPX4-centered antioxidant defenses but also by the abundance and subcellular distribution of polyunsaturated fatty acid (PUFA)-containing phospholipids in cellular membranes (19). ACSL4 plays a central role by preferentially activating *ω*-6 PUFAs such as arachidonic and adrenic acids to PUFA-CoA thioesters, thereby providing substrates for membrane lipid remodeling (39). Activated PUFAs are then selectively incorporated into specific phospholipid pools, with phosphatidylethanolamines (PEs) being a major acceptor class, enriching membranes with peroxidation-prone PUFA-phospholipids (39, 40). This substrate vulnerability is heightened during myocardial I/R, when oxidative load increases together with the labile iron pool. Lipid hydroperoxides can accumulate and compromise membrane integrity when GPX4-dependent detoxification is insufficient, accelerating ferroptotic injury (19, 40). Consistent with this model, ACSL4 upregulation generally correlates with increased lipid peroxidation and ferroptotic phenotypes, whereas ACSL4 downregulation through upstream signaling or ubiquitin-dependent degradation via E3 ubiquitin ligases can alleviate ferroptosis-related injury and improve I/R outcomes (41–43). Interaction between lipid remodeling, antioxidant defense, and iron handling during myocardial I/R is described in Figure 1.

## Regulation of iron homeostasis and the function of CMECs

### Mechanisms of iron uptake and storage

Iron homeostasis is a key determinant of CMEC performance during I/R, where rapid redox shifts can turn iron handling into a rate-limiting driver of lipid peroxidation (44). While many mechanistic frameworks for iron import, storage, and export during myocardial reperfusion have been established in cardiomyocytes and in generic endothelial systems, CMEC-specific evidence remains comparatively limited (44–47). In principle, endothelial iron uptake can proceed via transferrin (Tf)–transferrin receptor 1 (TfR1)-dependent endocytosis, followed by endosomal acidification/reduction and cytosolic delivery of iron to the labile iron pool (LIP), or via transferrin-independent pathways depending on the *in vivo* context (44–47). Notably, import routes are tightly coupled to storage, recycling, and export, so intracellular iron availability reflects coordinated regulation across these modules rather than a single transporter (44, 47).

The ubiquitin-specific protease 7 (USP7)-p53-TfR1 axis, which adapts to stress-induced reorganization of uptake machinery, is a representative example of this type of stress-induced reorganization during myocardial I/R, with USP7 mediating p53 deubiquitination associated with augmented TfR1 expression, iron build-up, lipid peroxidation, and ferroptosis-linked injury of the reperfused rat heart (45). The most common cellular source of these signals can be dependent on the model, but is consistent with the larger body of knowledge indicating that ubiquitin-dependent regulation can be detected at points of crossing with ferroptosis cues, and these points can be used to regulate iron-mediated oxidative damage through the added efforts of deubiquitinases other than their previously mentioned traditional functions in transcriptional regulation (46). It is worth noting that hypoxia-elicited CMEC-derived exosomal microRNA-210-3p (miR-210-3p) has been reported to alleviate hypoxia/reoxygenation-induced myocardial cell injury by inhibiting TfR1-mediated ferroptosis (12). On the experimental side, endocytic uptake inhibition is able to decrease iron uptake and, hence, curtail ferroptosis in cell models, which is an upstream pathway that is sensitive to modulation (47).

Simultaneously, storage systems buffer iron. Ferritin is a nanocage that sequesters surplus iron; each molecule can store up to ∼4,500 iron atoms, thereby mineralizing iron and limiting the toxicity of the labile iron pool (48). Ferritin stores can also be mobilized through ferritinophagy, a selective form of autophagy in which nuclear receptor coactivator 4 (NCOA4) delivers ferritin to lysosomes for degradation (49). Excessive or prolonged ferritin mobilization increases the LIP, promotes ROS generation, and sensitizes cells to lipid peroxidation and ferroptosis (50). Recent work further refined this model by showing that NCOA4 can undergo iron-dependent condensation, thereby selecting ferritin fates under iron-replete conditions and highlighting that ferritin turnover is a regulated process (51).

From the perspective of myocardial I/R, CMEC ferroptotic vulnerability may arise at the intersection of two iron fluxes: increased import via TfR1 and increased ferritin turnover via NCOA4 (45, 49–51). Mechanistically grounded strategies that attenuate pathological TfR1 upregulation and stabilize ferritin handling through NCOA4-mediated processes may help maintain microvascular endothelial viability and improve post-reperfusion microcirculatory outcomes (45, 49–51).

### Iron export and antioxidant capacity

**Figure 2:**
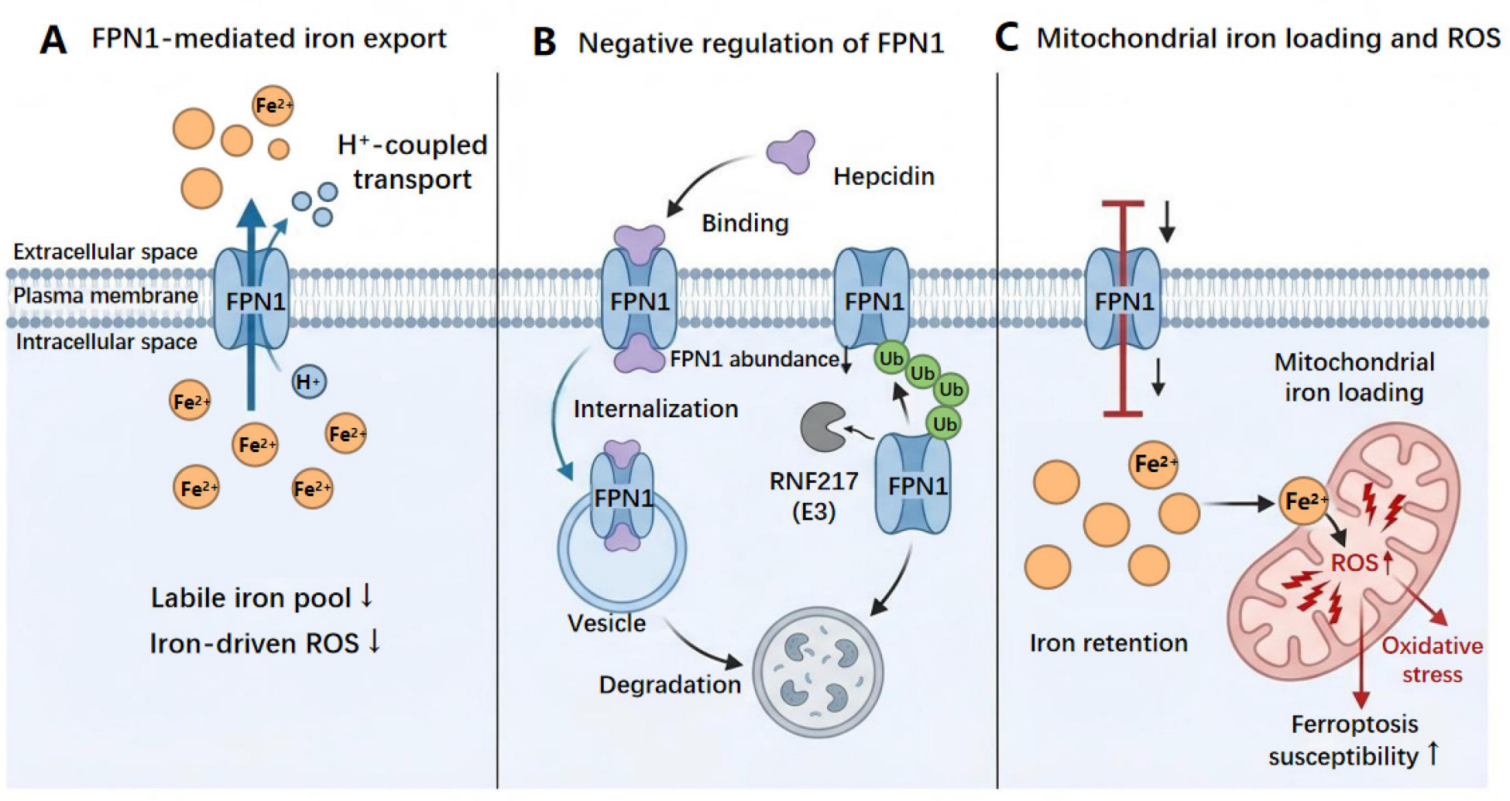
Regulation of FPN1-mediated iron export and mitochondrial redox consequences in cardiac microvascular endothelial cells during ischemia-reperfusion. **(A)** FPN1 exports ferrous iron (Fe^2^⁺) through proton-coupled transport, reducing the LIP and limiting iron-driven ROS production. **(B)** Hepcidin binding triggers FPN1 internalization and degradation, decreasing FPN1 abundance at the plasma membrane and thereby diminishing iron export. RNF217 (E3)-mediated ubiquitination further promotes FPN1 degradation. **(C)** Reduced FPN1 activity leads to intracellular iron retention and mitochondrial iron loading, increasing ROS generation and oxidative stress and elevating susceptibility to ferroptosis. FPN1, ferroportin 1; Fe²⁺, ferrous iron; H⁺, proton; ROS, reactive oxygen species; RNF217, ring finger protein 217; E3, ubiquitin ligase; Ub, ubiquitin. Symbols: ↑, increase; ↓, decrease.

In the case of I/R, reoxygenation quickly elevates oxidative pressure and lipid peroxidation, and access to iron is typically the factor that switches cells between reversible and ferroptotic injury (52–55). The known iron exporter of cells is Ferroportin 1 (FPN1) (52). FPN1 reduces the labile iron pool by exporting Fe²⁺, thereby limiting iron-catalyzed radical propagation and lipid peroxidation (52). The LIP has been generally considered one of the major factors which determine cell responsiveness to oxidative stress (53). There is microvascular endothelial work also demonstrating that FPN1 collaborates with exocytoplasmic ferroxidase action to facilitate iron removal out of endothelial barrier in brain microvascular endothelial models; thus, also demonstrating that endothelial iron export exists and is controllable (56). The generalization of this framework is in Figure 2A, which represents FPN1-mediated export along with proton-coupling.

One of the most potent suppressors of this protective pathway is hepcidin (57). Hepcidin binding triggers internalization and degradation of FPN1, inhibiting iron efflux and promoting intracellular iron retention. This process is reflected in Figure 2B (57). Beyond hepcidin, FPN1 stability is influenced by general cellular quality-control circuits (58, 59). Autophagy-related programs can remodel iron handling and distribution, although much of the systemic evidence derives from macrophage studies (58). More recently, ring finger protein 217 (RNF217) was identified as an E3 ubiquitin ligase that promotes FPN1 ubiquitination and subsequent degradation via a TET1-related regulator*y* axis; Figure 2B has been updated to include this hepcidin-independent negative module (59). Second, abundance measurements alone do not capture FPN1 flux (54). Human FPN1 is a proton-coupled transporter, suggesting that export efficiency may be modulated by transmembrane proton gradients during stress, consistent with the transport mode in Figure 2A (54).

These regulatory layers directly influence antioxidant competence because mitochondria are major sites of iron utilization (heme synthesis and Fe–S cluster biogenesis) and are highly sensitive to iron mishandling (55). Disrupted mitochondrial iron homeostasis increases electron leakage from the respiratory chain and elevates mitochondrial ROS, which weakens cellular antioxidant capacity and promotes lipid peroxidation (55). As summarized in Figure 2C, reduced FPN1 activity favors intracellular iron retention, mitochondrial iron loading, and ROS amplification, thereby increasing endothelial susceptibility to ferroptotic death (55). Overall, the hepcidin–FPN1 axis together with associated ubiquitin and transport systems regulates both labile and mitochondrial iron pools, offering a mechanistic explanation for how iron dyshomeostasis can heighten oxidative injury and ferroptotic risk during I/R (54–59).

## Experimental evidence of ferroptosis and microcirculatory injury

### Ferroptosis *in vitro* and animal models

*In vitro* H/R is widely used to mimic I/R stress and provides a practical window to interrogate how disturbed iron handling couples to lipid peroxidation (11, 60–62). In human CMECs, H/R compromises migration and tube formation and is accompanied by increased ROS and lipid peroxidation, mitochondrial ultrastructural injury, and weakened GPX4-associated antioxidant defense (11, 60–62). Erastin exacerbates these changes, whereas ferrostatin-1 (Fer-1) partially reverses both ferroptosis-associated injury and endothelial functional impairment, supporting a causal contribution of ferroptotic signaling to endothelial injury under H/R stress (11, 60, 62).

Beyond inhibiting lipid peroxidation, strategies that directly reduce the LIP are being explored. In a mouse I/R model, the iron chelator deferasirox reduces ferroptosis-associated injury and infarct size, and combination with cyclosporine A confers additional benefit, suggesting complementary effects between iron limitation and inhibition of mitochondrial permeability transition (63). Taken together, current evidence supports a more coherent chain of events in which iron dyshomeostasis during reoxygenation fuels lipid peroxidation and mitochondrial dysfunction, ultimately translating into microvascular endothelial injury and microcirculatory impairment (11, 60–63). Representative *in vitro* and *in vivo* evidence and key model features are summarized in Table 1.

**Table 1:** Representative experimental evidence linking ferroptosis in cardiac microvascular endothelial cells to ischemia-reperfusion-associated microcirculatory injury.

Model and subjects	Evidence of ferroptosis	Endothelial and microcirculatory readouts	Mechanistic and therapeutic clues	Ref
Exosomes derived from hypoxia-conditioned CMECs; co-evaluated with cardiomyocyte H/R	Ferroptosis-related changes accompanied by TfR1-associated signaling alterations	Downstream cardiomyocyte H/R injury phenotypes were altered in the exosome-transfer setting (microcirculatory perfusion was not directly assessed)	miR-210-3p-dependent regulation with inhibition of TfR1-associated ferroptotic processes. This supports an endothelial-cardiomyocyte paracrine axis relevant to I/R phenotypes	(12)
Human CMECs exposed to H/R	Increased lipid peroxidation and labile iron, reduced GPX4-associated defense, with mitochondrial ultrastructural damage; ferroptosis inhibition partially reverses changes	Impaired migration and tube formation, suggesting compromised endothelial repair and angiogenic capacity	ENPP2 overexpression mitigates H/R-associated ferroptosis; linked to PI3 K/AKT/mTOR signaling	(11)
Rat I/R with parallel validation in human CMECs under H/R	Elevated Fe²⁺, MDA and lipid peroxidation with downregulation of SLC7A11/GPX4; improved after intervention	Perfusion labeling and imaging indicate improved microvascular perfusion, accompanied by functional benefit	PEDF and its 34-mer peptide engage the Nrf2/HO-1 antioxidant axis, alleviating endothelial ferroptosis and improving perfusion	(13)
Rat I/R and CMECs under H/R	Ferroptosis-related markers and lipid peroxidation increased; GPX4 axis restored by treatment	Improved cardiac microvascular functional outcomes and attenuated I/R injury phenotypes	Naringin promotes mitochondrial translocation of NDUFS1 and activates IRF3/SLC7A11/GPX4 signaling to suppress endothelial ferroptosis	(14)
Mouse cardiac I/R with endothelial-specific genetic manipulation and reperfusion-phase microcirculatory assessment	Endothelial ferroptosis correlates with enhanced lipid peroxidation; GPX4 stability and phosphorylation state align with phenotypes	Reduced microvascular perfusion and increased no-reflow area; pathway activation or pharmacological stimulation improves perfusion and outcomes	sGC-cGMP-PKG signaling regulates LDHA- and GPX4-phosphorylation to limit GPX4 degradation and endothelial ferroptosis; vericiguat shows protection	(15)

CMECs, cardiac microvascular endothelial cells; I/R, ischemia-reperfusion; H/R, hypoxia/reoxygenation; TfR1, transferrin receptor 1; miR-210–3p, microRNA-210-3p; GPX4, glutathione peroxidase 4; ENPP2, ectonucleotide pyrophosphatase/phosphodiesterase 2 (autotaxin); PI3K, phosphoinositide 3-kinase; AKT, protein kinase B; mTOR, mechanistic target of rapamycin; Fe²⁺, ferrous iron; MDA, malondialdehyde; SLC7A11 (xCT), solute carrier family 7 member 11; PEDF, pigment epithelium-derived factor; 34-mer, 34-amino-acid peptide fragment; Nrf2, nuclear factor erythroid 2-related factor 2; HO-1, heme oxygenase-1; NDUFS1, NADH:ubiquinone oxidoreductase core subunit S1; IRF3, interferon regulatory factor 3; sGC, soluble guanylate cyclase; cGMP, cyclic guanosine monophosphate; PKG, protein kinase G (cGMP-dependent protein kinase); LDHA, lactate dehydrogenase A.

### Effects of ferroptosis-targeted interventions in animal models

Evidence from preclinical I/R models supports ferroptosis as a druggable contributor to acute injury and early remodeling (23, 64). Representative anti-ferroptotic interventions delivered around reperfusion show infarct-sparing and redox-protective effects in myocardial I/R settings (23, 64).

Beyond canonical radical-trapping antioxidants, several pathway-oriented interventions appear to converge on ferroptosis control. Isoliquiritigenin, for example, mitigated myocardial I/R injury while reinforcing the nuclear factor erythroid 2-related factor 2 (Nrf2)/heme oxygenase-1 (HO-1)/SLC7A11/GPX4 axis and suppressing lipid peroxidation, consistent with an anti-ferroptotic mode of protection *in vivo* (65). It is important to note that new-generation work is already setting the microvascular endothelium in this therapeutic perspective (13, 65). PEDF-derived 34-mer peptide enhanced cardiac microvascular perfusion measured by perfusion labeling/imaging, and this functional improvement was accompanied by reduced iron accumulation and lipid peroxidation markers of ferroptosis, along with restoration of SLC7A11 and GPX4 (13). Similar experiments in human CMECs further supported an endothelial mechanism by showing that Nrf2/HO-1 signaling mediates ferroptosis inhibition (13).

Existing animal evidence supports ferroptosis-focused approaches as a realistic strategy to safeguard myocardium and microcirculation, but also highlights practical gaps (13, 23, 64, 65). Priorities include defining therapeutic windows around reperfusion, quantifying microvascular endpoints alongside infarct metrics, and integrating endothelial-centered ferroptosis modulation with cardiomyocyte-centered approaches (13, 64).

Although evidence for ferroptosis in cardiac microvascular endothelial cells is strongest in cell-culture models of ischemia-reperfusion-like stress, publicly available single-cell transcriptomic datasets from post-ischemic hearts (66, 67) now make it possible to interrogate iron metabolism and ferroptosis-associated gene programs *in vivo* with endothelial resolution. Single-cell RNA-seq resources spanning multiple reperfusion stages contain endothelial clusters in which iron import and export machinery, heme- and iron-stress response pathways, and antioxidant control nodes can be examined at single-cell scale, thereby complementing bulk-tissue measurements (66, 67). These datasets cannot, on their own, demonstrate that ferroptotic execution has occurred, but they can pinpoint when and where cardiac microvascular endothelial cells adopt a pro-ferroptotic state, reflected by impaired iron handling together with attenuation of GPX4-centered protective capacity (66, 67). They can also guide focused validation in reperfused microvessels using endothelial lipid-peroxidation reporters and probes for the labile iron pool (66, 67).

To obtain causal evidence for ferroptotic activity in cardiac microvascular endothelium *in vivo*, genetic strategies are especially informative (37, 38). Conditional alleles of core ferroptosis regulators [e.g., GPX4, SLC7A11, or AIFM2 (also known as FSP1)] can be interrogated using inducible endothelial Cre systems, including widely used pan-endothelial drivers such as VE-cadherin or Cdh5, to test whether endothelial-specific sensitization to ferroptosis—or reinforcement of anti-ferroptotic protection—alters microvascular obstruction, vascular leakage/edema, or the no-reflow phenomenon following myocardial I/R (37, 38). In parallel, iron-handling pathways (e.g., ferritinophagy control nodes upstream of iron release) can be manipulated to increase iron mobilization and clarify whether endothelial iron release is rate limiting for injury (37, 38, 49–51). These causal interpretations require careful assessment of recombination efficiency, cell-type specificity, heterogeneity across microvascular segments, and rigorous measurement of endothelial outcomes together with infarct size.

## Integration of the mechanistic chain linking iron homeostasis dysregulation, ferroptosis, and microcirculatory injury

### Dysregulated iron homeostasis promotes lipid peroxidation and mitochondrial dysfunction

Altered iron homeostasis is a predisposing process that alters CMECs towards ferroptotic damage (22, 68, 69). Expansion of the intracellular labile ferrous iron pool enhances Fenton chemistry, generating highly reactive radicals and increasing ROS (22, 68, 69). The resulting oxidative stress propagates lipid peroxidation and accelerates lipid peroxide accumulation (22, 68, 69). Lipid peroxidation does not just represent a product of oxidative stress, but it is a key biochemical pathway that triggers ferroptotic development (22, 68). In cases where the lipid peroxide detoxification capacity is overwhelmed or physically unable to lower the lipid hydroperoxides, lipid hydroperoxides are not properly lowered, exacerbating membrane damage and reducing endothelial barrier capacity (22, 68, 69).

Increased influx is not the sole determinant in iron overload. Forced ferritinophagy through NCOA4 helps to mobilize iron stored in the cell, increases the size of the LIP, and amplifies iron-mediated oxidative amplification (50). This path can further be hastened by inflammatory signals. It has been reported that interleukin-6 (IL-6) favors the effect of ROS-dependent lipid peroxidation and disturbs cellular iron homeostasis, making them prone to ferroptosis (70). From the perspective of systemic iron regulation, IL-6 signaling via signal transducer and activator of transcription 3 (STAT3) induces hepcidin expression, which suppresses ferroportin-mediated iron export. This favors intracellular iron retention and links inflammation to cellular iron loading and oxidative lipid damage (71–73).

The center of cellular bioenergetics and a significant location of iron usage are the mitochondria, where iron-sulfur cluster biogenesis is predominantly accumulated, and the heme synthesis happens (74, 75). To this end, the iron dyshomeostasis shifting iron to the mitochondrial deposition direction is frequently coupled with the enhanced production of ROS, damage of the mitochondrial membrane, respiratory chain dysfunction, and the decreased ATP production, which essentially transforms oxidative stress into energy deficit (75–77). Cardiovascular research also suggests that oxidative stress is capable of facilitating mitochondrial iron overload and ferroptosis-like cell damage into cardiomyocytes (76). Consistent with findings in hypoxia/reoxygenation models of CMECs, ferroptosis has been attributed to both mitochondrial dysfunction and deteriorated migratory and angiogenic capacity (11). Moreover, disturbed mitochondrial dynamics and defective mitophagy can reciprocally amplify ferroptosis under sustained stress, sustaining ROS and lipid peroxidation in a self-reinforcing cycle (77).

Taken together, labile iron expansion and mitochondrial iron misdistribution provide a catalytic foundation for lipid peroxidation and drive CMEC ferroptosis through convergent membrane injury and bioenergetic failure, offering a coherent cellular link to microvascular dysfunction during reperfusion (11, 22, 68–77).

### Ferroptosis-induced endothelial barrier disruption and increased permeability

**Figure 3:**
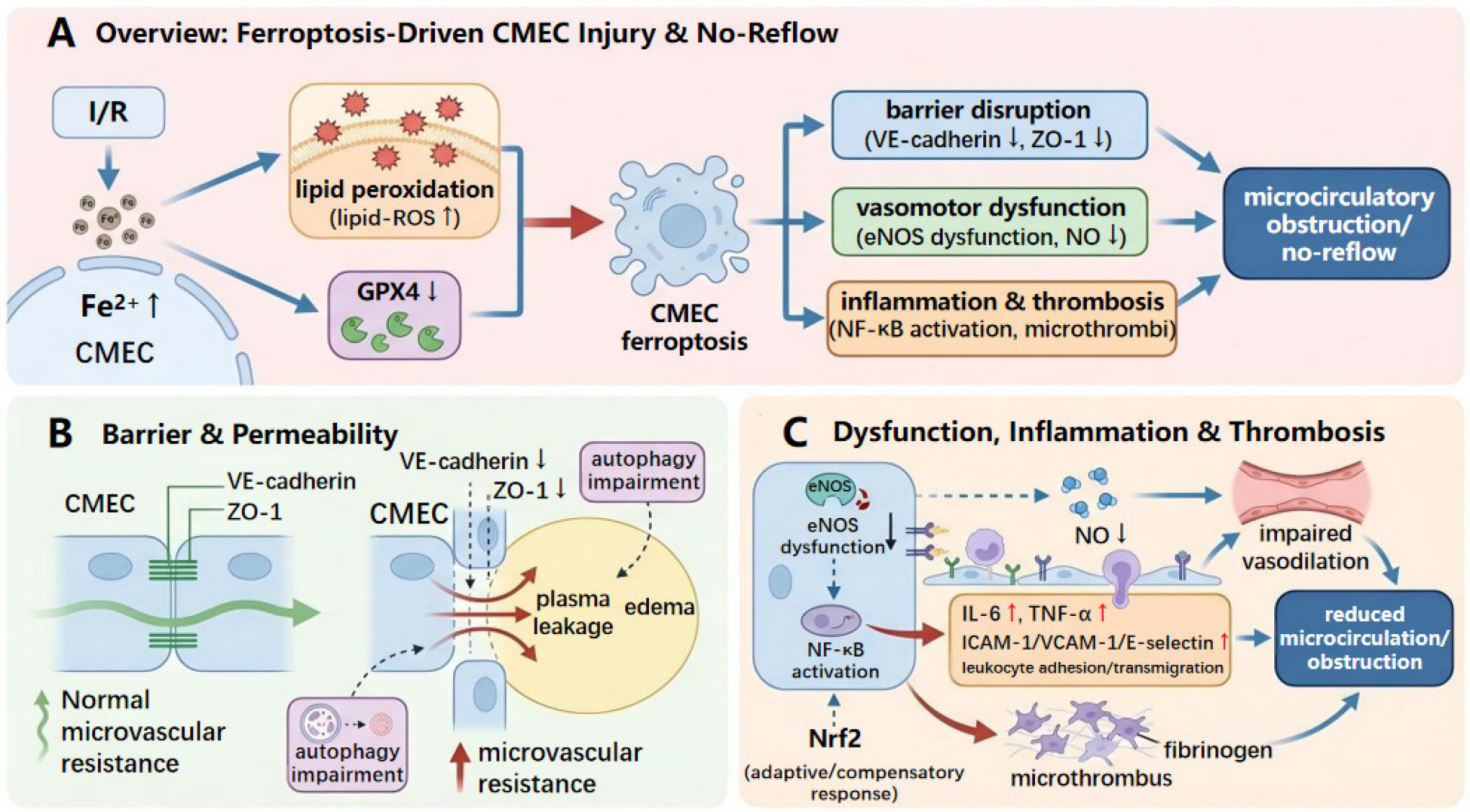
Ferroptosis-driven cardiac microvascular endothelial injury promotes microcirculatory obstruction and no-reflow during myocardial ischemia-reperfusion. Proposed CMEC-centered model linking dysregulated iron handling to ferroptosis-driven microcirculatory injury during myocardial ischemia-reperfusion. Reperfusion-associated ROS and iron mobilization expand the LIP and promote phospholipid peroxidation, lowering the ferroptosis threshold in CMECs. Ferroptotic and sublethally injured CMECs impair junctional integrity, increase permeability and edema, amplify inflammatory cell adhesion, and contribute to capillary obstruction and no-reflow, thereby worsening tissue hypoxia and downstream cardiomyocyte injury. Therapeutic nodes include limiting reactive iron, blocking lipid radical propagation, and reinforcing antioxidant defense pathways to preserve microvascular function. CMEC, cardiac microvascular endothelial cell; I/R, ischemia-reperfusion; ROS, reactive oxygen species; LIP, labile iron pool; GPX4, glutathione peroxidase 4.

In the context of I/R, CMECs develop iron dyshomeostasis and an increased lipid peroxidation burden (14, 78). Rather than serving as a generic oxidative by-product, ferroptotic lipid peroxidation directly threatens endothelial membrane stability and junctional integrity—two determinants of microvascular leakage, edema, leukocyte/platelet plugging, and thereby potentially contributing to the no-reflow phenotype (14). As shown in Figure 3, iron overload promotes lipid-ROS accumulation and destabilizes membrane architecture, whereas compromised GPX4 activity weakens lipid-peroxide detoxification and accelerates barrier failure (14, 78). Importantly, barrier/junction-focused CMEC-specific causal validation *in vivo* remains limited, underscoring the need for endothelial-targeted, time-resolved studies using microvascular endpoints (78–83).

Cardiac-specific work underscores the centrality of microvascular barrier integrity during reperfusion and provides heart-relevant endothelial endpoints that can be paired with ferroptosis markers (79). In a rat I/R model, the endothelial protectant CU06-1004 preserved VE-cadherin and zonula occludens-1 (ZO-1), reduced edema and inflammatory activation, and improved subsequent remodeling, highlighting junction integrity as a clinically relevant target in the heart (79).

Evidence from non-cardiac microvascular endothelial models, including studies in the brain and spinal cord, suggests that ferroptosis can accompany loss of junctional proteins and barrier breakdown, and that ferroptosis inhibition can partially restore permeability control (78, 80). However, whether CMECs follow the same junctional sequence during I/R remains to be established (78, 80). Future work should therefore use CMEC-targeted, time-resolved *in vivo* approaches that assess VE-cadherin and ZO-1 together with microvascular leakage, edema, perfusion, and no-reflow outcomes.

The relationship between junctional disruption and ferroptosis is likely bidirectional rather than merely correlative (78, 81, 82). Iron-driven phospholipid peroxidation can compromise membrane integrity and cytoskeletal anchoring, leading to internalization, shedding, or proteolytic processing of junctional components and thereby increasing vascular permeability before frank cell death occurs (78, 81, 82). Conversely, loss of junctional organization may heighten endothelial vulnerability to ferroptotic stress by reshaping mechanotransduction, disturbing redox balance, and altering the composition and spatial organization of membrane lipids (78, 81–83). Consistent with this idea, work in other microvascular endothelial settings indicates that ferroptosis inhibition can partly preserve tight and adherens junction proteins and maintain barrier function, while disruption of VE-cadherin–dependent signaling can promote phenotypes consistent with ferroptotic injury (78, 81, 82).

In cardiac microvascular endothelial cells, demonstrating causality will require experimental designs that separate junctional failure from lipid peroxidation. One approach is to stabilize junctions while leaving iron availability and lipid reactive oxygen species largely unchanged, and to inhibit ferroptosis while independently tracking junctional turnover using genetic or reporter-based readouts. Integrating these manipulations with live measurements of permeability and lipid peroxidation within the same microvascular segments across reperfusion would help define the directionality, timing, and mechanistic coupling between barrier breakdown and ferroptotic signaling (83).

Beyond direct membrane injury, lipid peroxidation can also drive the activation of inflammation-related signaling and further weaken endothelial junction stability, thereby amplifying permeability abnormalities (84). At the same time, cytoskeletal tension and remodeling of intercellular junctions also participate in the regulation of permeability (83). Available evidence suggests that RhoA-related signaling can couple with ferroptotic processes and promote increased vascular permeability (85). In addition, autophagy dysfunction alters endothelial protein homeostasis related to junction maintenance; iron overload can exacerbate the degradation of junction proteins and further disrupt barrier structure (86). Collectively, lipid peroxidation, cytoskeletal remodeling, and impaired junction maintenance are expected to increase microvascular permeability, promote tissue edema, and raise microcirculatory resistance, predisposing to hypoperfusion and the no-reflow phenotype during early reperfusion (84–86). In CMECs, a practical definition of the ferroptotic execution state is PUFA-phospholipid peroxidation that exceeds GPX4-mediated detoxification in the presence of an expanded LIP (19, 22, 25). This biochemical state is expected to destabilize junctional complexes, blunt nitric oxide-dependent vasoreactivity, and favor an adhesive and prothrombotic endothelial surface, thereby amplifying leukocyte and platelet plugging and microthrombus formation (14, 84–86).

Beyond being a microvascular hallmark, endothelial swelling and barrier leakage can be upstream drivers of cardiomyocyte loss (2–4). Interstitial edema increases diffusion distance for oxygen and metabolites, raises extravascular compressive forces on capillaries, and aggravates perfusion heterogeneity, thereby prolonging functional ischemia even after epicardial revascularization (4). This creates a feed-forward loop in which microvascular leak and no-reflow expand the zone of persistent hypoxia, promote metabolic collapse in adjacent cardiomyocytes, and ultimately enlarge infarct size (2, 3). Encouragingly, several lines of evidence support reversibility of these phenotypes (11, 79). Pharmacologic endothelial barrier stabilization has been reported to reduce edema and improve microvascular perfusion/no-reflow indices in myocardial I/R models (79), while *in vitro* studies in human CMECs show that ferroptosis inhibition can restore endothelial functional readouts under hypoxia/reoxygenation (including migration and angiogenic capacity) (11). Together, these observations support targeting endothelial ferroptosis and barrier failure as a microcirculation-directed strategy to limit secondary cardiomyocyte injury during reperfusion (11, 79).

### Ferroptosis exacerbates endothelial dysfunction and inflammatory responses

Other than disrupting barriers, newly discovered cardiac markers implicate CMEC ferroptosis in inhibited microvascular perfusion and growth of the no-reflow domain during reperfusion, specifically in models where endothelial-directed manipulation and no-reflow area should be evaluated (15). Simultaneously, a state of ferroptosis-related oxidative damages alongside low migration and tube formation in human CMECs under H/R signifies impaired endothelial repair ability (11). These results underpin the active role of the CMEC ferroptosis in microcirculatory dysfunction and downstream programs, including vasomotor, inflammatory, and prothrombotic program switching, remain to be validated directly using CMEC-targeted approaches in the heart models (11, 15). Mechanistically, the lipid peroxidation of iron can support oxidative stress and impair the lipid peroxide detoxification process mediated by GPX4, which in turn lays the basis to resist endothelial dysfunction at the onset of reperfusion (14, 84).

Endothelial vasomotor control may also be impaired (84, 87). Oxidative stress has been linked to decreased endothelial nitric oxide synthase activity, reduced bioavailability of nitric oxide, and dysfunctional vasodilatory responses in endothelial models that may subsequently restrict microcirculatory perfusion (84, 87). Nuclear factor-*κ*B (NF-*κ*B)-related inflammatory activation, augmented discharge of pro-inflammatory mediators, and augmented expression of adhesion products have also been correlated with oxidative stress and lipid peroxidation products (84). These alterations can favor leukocyte adhesion and transendothelial migration, enhance inflammatory infiltration, and augment microvascular resistance, and adhesion molecules like intercellular adhesion molecule-1 (ICAM-1), vascular cell adhesion molecule-1 (VCAM-1), E-selectin are often found to be increased in that environment (84). These downstream programs in cardiac CMEC-targeted models should also be confirmed directly (84, 87–89).

Under sustained oxidative and inflammatory stress, platelet adhesion and activation may become more likely, increasing the risk of microthrombus formation and aggravating microcirculatory obstruction, which could contribute to no-reflow (88, 89). In addition, iron overload-related dysregulation of autophagy has been reported to promote degradation of junctional proteins such as VE-cadherin and ZO-1, which may further worsen barrier disruption and sustain perfusion restriction during I/R (86).

### Crosstalk and synergistic interactions between ferroptosis and other forms of cell death

Oxidative stress lies upstream of many regulated cell-death pathways, but ferroptosis is distinguished by its reliance on iron-catalyzed phospholipid peroxidation and by failure of specific antioxidant control nodes (19, 22, 25). Key defenses involve cystine uptake through system x_c⁻ (the cystine/glutamate antiporter, xCT/SLC7A11), maintenance of the glutathione pool, and GPX4 activity, together with broader radical-trapping capacity (19, 22, 25). A ferroptosis-centered interpretation of cardiac microvascular endothelial cell injury is therefore most convincing when endothelial changes meet practical operational benchmarks (19, 22, 25). There is an increase in reactive or labile iron alongside heightened phospholipid peroxidation (19, 22, 25). GPX4-based protective capacity is reduced or demonstrably insufficient in functional terms (19, 22, 25). Protection is achieved by iron chelators or radical-trapping ferroptosis inhibitors rather than by agents that do not address ferroptotic mechanisms (11, 19, 22, 25). In hypoxia/reoxygenation models of cardiac microvascular endothelium, these benchmarks are increasingly observed together with functional deficits such as barrier breakdown and impaired angiogenic competence, supporting ferroptosis as a mechanistically grounded link between iron imbalance and microvascular failure (11).

Crosstalk among regulated death programs matters for two reasons (35, 36, 90–94). Reperfusion injury progresses through stages and varies across the microvasculature, so different death programs may predominate at different times and within different vascular segments. Focusing on only one pathway can therefore capture only a subset of endothelial loss (35, 36, 90–94). Therapeutic perturbations can also reshape the death landscape (35, 36, 90–94). Partial inhibition of one modality may reroute signaling into alternative death routes or intensify inflammatory escalation, reducing efficacy and sometimes producing counterintuitive outcomes (35, 36, 90–96). Therefore, mapping how ferroptosis interfaces with apoptosis, pyroptosis, necroptosis, and autophagy, through shared triggers (ROS, Ca²⁺ dysregulation, mitochondrial failure), shared inflammatory outputs, including damage-associated molecular patterns (DAMPs) and cytokines, and shared microvascular endpoints (permeability, edema, no-reflow) is necessary to interpret inconsistent drug responses and to design rational combination strategies that truly preserve CMEC viability and microvascular function during reperfusion (90, 91).

Oxidative stress and lipid peroxidation represent a shared hub (90). ROS and oxidized membrane lipids not only propel ferroptotic damage, but also intensify endoplasmic reticulum stress and proteostasis disruption, thereby lowering the threshold for mitochondrial instability and apoptotic signaling (90, 91). At the organelle level, mitochondria have been shown both to amplify redox deregulation and to be a location where multiple death signals may converge; mechanistic studies on mitochondrial membrane-proximal events have demonstrated tractable interactions between ferroptosis and apoptosis (92). Regularly, the evidence to incriminate a BCL2-associated X protein (BAX)-dependent mitochondrial pathway demonstrates that ferroptosis and apoptosis may be additive when it comes to anomaly programs on a specific stress intensity and schedule (93).

This coupling can be further increased by regulatory gates p53 is capable of extensively remodeling cystine metabolism and antioxidant ability, addressing ferroptosis vulnerability, indirectly adjusting the apoptotic threshold, which render pathway dominance crucially context dependent (94). This dependence on context is especially important to I/R, in which the first layer of reperfusion phases oxidative burst, ionic imbalance and inflammatory signaling leading to the concomitant activation of multiple death programs (35, 36).

The ferroptosis–pyroptosis interface may link regulated cell death to sterile inflammation and microvascular remodeling (95, 96). Progressive membrane damage and lipid peroxidation can trigger danger-signal release and feed into inflammasome-related cascades, increasing the likelihood and magnitude of pyroptotic amplification (95, 96). It is important to note that oligomerization of nerve injury-induced protein 1 (NINJ1), a cell-surface (plasma membrane) protein, can occur during ferroptosis and may represent an execution-level transition between membrane damage and inflammatory spread (97). At the microcirculatory level, these interactions are unlikely to be neutral (35, 36, 95–97). CMECs are exposed to iron dyshomeostasis, lipid oxidative stress and inflammatory mediators at reperfusion, and the convergence of multiple death programs can plausibly translate into barrier disruption, leukocyte recruitment, prothrombotic tendency and impaired vasoreactivity, thereby increasing microvascular resistance and worsening hypoperfusion and no-reflow-related injury (35, 36).

## Potential therapeutic strategies and prospects for clinical translation

### Ferroptosis inhibitors and agents regulating iron homeostasis

Ferroptosis is increasingly viewed as a tractable therapeutic node in I/R injury, where lipid peroxidation at reperfusion intersects with dynamic iron handling and compromised antioxidant defenses (23, 98, 99). Current preclinical strategies broadly follow two complementary directions, directly limiting lipid radical propagation and reshaping the LIP that sustains oxidative membrane injury (23, 98, 99).

Radical-trapping antioxidants remain the most commonly used ferroptosis inhibitors in cardiac ischemic settings. Puerarin protected against myocardial ischemia-reperfusion injury and was associated with suppression of ferroptosis-related changes, including reduced lipid peroxidation and restoration of GPX4 expression, supporting an anti-ferroptotic cardioprotective effect *in vivo* and *in vitro* (64). A second representative scaffold, Lip-1, reduced infarct size and preserved mitochondrial integrity when administered at the onset of reperfusion in isolated perfused mouse hearts, accompanied by restoration of GPX4 and modulation of voltage-dependent anion channel 1 (VDAC1) (23).

It should be noted that the apparent efficacy of ferroptosis inhibitors can vary across experimental settings, reflecting differences in pharmacokinetics, dosing, timing relative to reperfusion, and the dominant target cell population (23, 64, 98–100). Ferrostatin-1 (Fer-1), while widely used as a reference radical-trapping antioxidant, has recognized limitations in stability and bioavailability (100), and inconsistent protection in some *in vivo* contexts does not necessarily negate a role for ferroptosis but may indicate suboptimal target engagement in the relevant microvascular compartment. In addition, ferroptosis-associated signaling can be cell type- and stage-dependent; thus, indiscriminate or prolonged systemic anti-ferroptotic therapy could produce mixed effects on acute injury vs. later repair programs (23, 98–101). These considerations further argue for microvascular- and time-biased approaches to preferentially protect CMEC function while minimizing unintended effects in other cardiac cell types (23, 98–101). Consistent with this stage- and model-dependence, in a permanent left anterior descending (LAD) coronary artery occlusion myocardial infarction model in neonatal and juvenile mice, systemic Fer-1 was reported not to further reduce residual scarring, and in the non-regenerative stage it further decreased ejection fraction (EF) and fractional shortening (FS); the authors proposed that ferroptotic cardiomyocytes can support wound healing by releasing pro-angiogenic factors and reshaping immune responses (101).

Agents that regulate iron homeostasis provide a mechanistically aligned lever, though with greater systemic liability (99, 102, 103). Iron chelation is the most direct approach (99, 103). Deferoxamine (DFO) is frequently used experimentally to reduce the LIP and downstream ROS-driven injury, and has also served as a component of multi-target reperfusion regimens (99). Notably, ponatinib combined with DFO provided greater protection than either agent alone in ischemic heart injury models, consistent with concurrent suppression of necroptotic and ferroptotic components of reperfusion-associated damage (99). Beyond chelation, modulating iron trafficking has attracted attention, including the hepcidin-FPN1 axis (102, 103). In myocardial I/R, suppressing hepcidin was associated with reduced intramyocardial hemorrhage and adverse remodeling, supporting the concept that avoiding iron trapping may limit secondary oxidative injury (102). For clarity, the key ferroptosis inhibitors and iron homeostasis-modulating approaches discussed above, together with their mechanistic targets, development stage, and translational constraints, are summarized in Table 2.

**Table 2:** Representative ferroptosis-targeted and iron-handling strategies in myocardial ischemic injury models.

Intervention	Core anti-ferroptotic action	Representative evidence in myocardial ischemic settings	Translational considerations	Ref
Puerarin	Attenuates lipid peroxidation and ferroptosis-associated injury; restores GPX4-related antioxidant defense	In mouse myocardial I/R and cardiomyocyte OGD/R models, puerarin reduced myocardial injury and ferroptosis-related readouts, including MDA/4-HNE and Ptgs2, while increasing GPX4 expression	Natural product candidate; peri-reperfusion dosing, exposure, and reproducibility need further study	(64)
Lip-1	Radical-trapping antioxidant; suppresses lipid peroxidation and mitochondrial oxidative stress	In isolated perfused mouse hearts, dosing at reperfusion onset reduced infarct size and preserved mitochondria; restored GPX4 and reduced VDAC1 levels and oligomerization	Preclinical; requires delivery and timing optimization; durability and safety beyond acute dosing remain undefined	(23)
DFO and other iron chelators	Chelation reduces the LIP and downstream ROS-driven lipid peroxidation	Iron chelation reduced injury in experimental I/R and hypoxia/reoxygenation settings; frequently used as a component of multi-target regimens	DFO is clinically used for systemic iron overload, but cardiac I/R use would require carefully controlled dosing, timing, and safety monitoring	(99, 103)
Hepcidin-FPN1 axis modulation	Rebalances iron export and storage programs to reduce pathological iron trapping	In a mouse myocardial I/R model, suppressing cardiac hepcidin was linked to less intramyocardial hemorrhage and adverse remodeling	Systemic iron regulation raises off-target risk; patient selection and iron-status stratification are likely necessary for translation	(102, 103)
Ponatinib + DFO (combination)	Concurrently suppresses necroptotic and ferroptotic components of reperfusion injury	Provided greater protection than monotherapy in ischemic heart injury models while suppressing necroptosis and ferroptosis	Repurposing constraints include drug-drug interactions, dosing complexity, and safety considerations for ponatinib in acute cardiac settings	(99)
Allicin via biomimetic nanoparticles	Improves delivery to injured myocardium and limits ferroptosis-associated endothelial damage	Neutrophil membrane-camouflaged nanoparticles improved regional perfusion and cardiac function in I/R rats; inhibited cardiac microvascular endothelial ferroptosis and increased PECAM-1 expression	Manufacturing reproducibility, scale-up, and regulatory characterization are central barriers; benefits should be benchmarked against standard reperfusion care	(104)
tFNA-Cur (framework nucleic acid-curcumin complex)	Carrier-enabled antioxidant and anti-ferroptotic therapy; reinforces KEAP1-Nrf2 signaling and GPX4/HO-1 defenses	In rat I/R injury, reduced infarct size and Fe²⁺ accumulation and suppressed lipid peroxidation; linked to KEAP1-Nrf2 signaling and reduced mitochondrial ROS	Formulation and batch consistency, biodistribution, and acute safety profiling are key for clinical translation	(105)

GPX4, glutathione peroxidase 4; I/R, ischemia-reperfusion; OGD/R, oxygen-glucose deprivation/reoxygenation; MDA, malondialdehyde; 4-HNE, 4-hydroxynonenal; Ptgs2, prostaglandin-endoperoxide synthase 2; Lip-1, liproxstatin-1; DFO, deferoxamine; ROS, reactive oxygen species; SLC7A11 (xCT), light chain of the system x_c⁻, cystine/glutamate antiporter; VDAC1, voltage-dependent anion channel 1; FPN1, ferroportin 1; PECAM-1, platelet endothelial cell adhesion molecule-1 (CD31); KEAP1, Kelch-like ECH-associated protein 1; Nrf2, nuclear factor erythroid 2-related factor 2; HO-1, heme oxygenase-1; Fe²⁺, ferrous iron; tFNA-Cur, tetrahedral framework nucleic acid-curcumin complex.

Clinical translation will likely depend on defining the most relevant target, the key cell population, and the narrow therapeutic window around early reperfusion (23, 98, 99). Many candidate agents act broadly on redox biology or systemic iron handling, which increases off-target concerns if exposure extends beyond the acute phase (23, 98–103). Delivery systems that bias exposure toward the ischemic microvasculature and the reperfusion stage, together with biomarker-guided timing, may ultimately determine whether anti-ferroptotic therapy can complement contemporary reperfusion care (23, 98, 99).

From a microcirculation-first perspective, anti-ferroptotic strategies that center on cardiac microvascular endothelial cells should treat preservation of barrier integrity and maintenance of capillary patency as primary outcomes, rather than relying on infarct size alone (2–4). One practical direction is to strengthen endothelial antioxidant defenses, including the cystine import–glutathione–GPX4 network and other lipid radical–buffering systems, so that lipid peroxidation remains below the threshold that destabilizes junctions (79, 102, 103). A second direction is to reduce endothelial exposure to reactive iron at the moment of reperfusion, with particular attention to conditions in which intramyocardial hemorrhage or heme and iron release are prominent (102, 103). A third direction is to pair ferroptosis inhibition with interventions that stabilize the endothelial barrier and limit edema, while also dampening leukocyte and platelet adhesion, thereby addressing both endothelial loss and the no-reflow component of microvascular injury (79).

Drug-delivery approaches that preferentially increase exposure within the reperfused microvasculature may further improve target engagement in cardiac microvascular endothelium while minimizing systemic effects (102, 103). Examples include carriers engineered to accumulate in endothelium and affinity-based targeting strategies that recognize luminal endothelial markers (102, 103). Future preclinical studies should therefore evaluate ferroptosis-related readouts alongside microvascular function measurements, including vascular leak, edema burden, perfusion heterogeneity, and capillary obstruction with no-reflow, to determine whether endothelial-selective protection provides added benefit beyond therapies that primarily target cardiomyocytes (2, 3).

### Natural products and combination therapy

Natural products are attractive because they can engage multiple converging processes relevant to ferroptosis, including redox buffering, inflammatory signaling, and endothelial function (23, 98, 99). At the same time, pleiotropic mechanisms complicate target engagement and dose-exposure relationships, which has fueled strategies pairing natural compounds with delivery technologies and rational combinations designed for time-critical reperfusion injury (99).

Allicin provides an example with microcirculatory relevance (104). Using neutrophil membrane-camouflaged biomimetic nanoparticles to enhance delivery to injured myocardium, allicin reduced infarct size and improved regional perfusion in an I/R rat model (104). The study also reported reduced ferroptotic injury in CMECs and increased PECAM-1 expression, consistent with protection of endothelial integrity and microvascular perfusion (104). Curcumin is another extensively researched candidate but its translational capability is hindered by solubility and tissue exposure (105). Curcumin has limited translational potential due to poor solubility and bioavailability (105). Formulation strategies, such as framework nucleic acid–curcumin complexes, may enhance delivery and anti-ferroptotic efficacy via KEAP1–Nrf2 signaling (105).

It is now being considered to complement the different cell-death responses of I/R with combination therapy, with Ponatinib combined with DFO demonstrating greater cardioprotection than monotherapy suggesting that simultaneous inhibition of necroptosis and ferroptosis can be effectively applied to reperfusion-related injury (99). The remaining practical challenge is to specify combinations that preserve mechanistically coherent and yet clinically implementable combination of drugs, and drug-drug interactions and dose schedules that can be handled as acute reperfusion workflows (98, 99).

## Conclusion

Restoring capillary-level reperfusion remains the central challenge beyond reopening the epicardial artery (2–4). The evidence summarized here supports a working model in which iron dysregulation lowers the ferroptosis threshold in CMECs and thereby contributes to microvascular barrier failure and impaired reperfusion during early I/R (11, 13–15). Key priorities now include endothelial-specific, time-resolved *in vivo* validation with rigorous microvascular endpoints, and defining therapeutic windows and delivery strategies that preferentially target the reperfused microvasculature (23, 63–65, 79, 102–105).

Although ferroptosis inhibition and iron-modulating strategies show benefit in preclinical I/R models, endothelial-specific and time-resolved *in vivo* studies are still needed to establish causality, define the therapeutic window, and delineate interactions with parallel death programs (23, 63–65, 99, 102–105). Future translational progress will likely depend on integrating microvasculature-targeted delivery with biomarker-guided timing and rigorous microvascular endpoints (79, 102–105). Such an approach may complement contemporary reperfusion care by preserving endothelial viability, improving tissue-level reperfusion, and mitigating no-reflow-associated myocardial injury (2–4, 11, 13, 15, 79).

## References

[1] Hausenloy DJ Yellon DM. Myocardial ischemia-reperfusion injury: a neglected therapeutic target. J Clin Invest. (2013) 123(1):92–100. 10.1172/JCI62874 23281415 PMC3533275

[2] Bouleti C Mewton N Germain S. The no-reflow phenomenon: state of the art. Arch Cardiovasc Dis. (2015) 108(12):661–74. 10.1016/j.acvd.2015.09.006 26616729

[3] Jaffe R Dick A Strauss BH. Prevention and treatment of microvascular obstruction-related myocardial injury and coronary no-reflow following percutaneous coronary intervention: a systematic approach. JACC. (2010) 3(7):695–704. 10.1016/j.jcin.2010.05.004 20650430

[4] Zhao B-H Ruze A Zhao L Li Q-L Tang J Xiefukaiti N The role and mechanisms of microvascular damage in the ischemic myocardium. Cell Mol Life Sci. (2023) 80(11):341. 10.1007/s00018-023-04998-z 37898977 PMC11073328

[5] Sorop O Van De Wouw J Merkus D Duncker DJ. Coronary microvascular dysfunction in ischaemic heart disease: lessons from large animal models. Basic Clin Pharmacol Toxicol. (2025) 137(2):e70074. 10.1111/bcpt.70074 40631421 PMC12239061

[6] Konst RE Guzik TJ Kaski J-C Maas AHEM Elias-Smale SE. The pathogenic role of coronary microvascular dysfunction in the setting of other cardiac or systemic conditions. Cardiovasc Res. (2020) 116(4):817–28. 10.1093/cvr/cvaa009 31977015 PMC7526753

[7] Fang X Ardehali H Min J Wang F. The molecular and metabolic landscape of iron and ferroptosis in cardiovascular disease. Nat Rev Cardiol. (2023) 20(1):7–23. 10.1038/s41569-022-00735-4 35788564 PMC9252571

[8] Xie L-H Fefelova N Pamarthi SH Gwathmey JK. Molecular mechanisms of ferroptosis and relevance to cardiovascular disease. Cells. (2022) 11(17):2726. 10.3390/cells11172726 36078133 PMC9454912

[9] Zhang Y Xin L Xiang M Shang C Wang Y Wang Y The molecular mechanisms of ferroptosis and its role in cardiovascular disease. Biomed Pharmacother. (2022) 145:112423. 10.1016/j.biopha.2021.112423 34800783

[10] Chen Y Li X Wang S Miao R Zhong J. Targeting iron metabolism and ferroptosis as novel therapeutic approaches in cardiovascular diseases. Nutrients. (2023) 15(3):591. 10.3390/nu15030591 36771298 PMC9921472

[11] Fang G Shen Y Liao D. ENPP2 Alleviates hypoxia/reoxygenation injury and ferroptosis by regulating oxidative stress and mitochondrial function in human cardiac microvascular endothelial cells. Cell Stress Chaperones. (2023) 28(3):253–63. 10.1007/s12192-023-01324-1 37052764 PMC10167086

[12] Lei D Li B Isa Z Ma X Zhang B. Hypoxia-elicited cardiac microvascular endothelial cell-derived exosomal miR-210-3p alleviate hypoxia/reoxygenation-induced myocardial cell injury through inhibiting transferrin receptor 1-mediated ferroptosis. Tissue Cell. (2022) 79:101956. 10.1016/j.tice.2022.101956 36272206

[13] Lu P Qi Y Li X Zhang C Chen Z Shen Z PEDF And 34-mer peptide inhibit cardiac microvascular endothelial cell ferroptosis via Nrf2/HO-1 signalling in myocardial ischemia-reperfusion injury. J Cell Mol Med. (2024) 28(14):e18558. 10.1111/jcmm.18558 39048917 PMC11269049

[14] Guan X Yang Z Wang J Lu W Wang S Yang M Naringin attenuates myocardial ischemia-reperfusion injury by promoting mitochondrial translocation of NDUFS1 and suppressing cardiac microvascular endothelial cell ferroptosis. J Nutr Biochem. (2025) 145:110019. 10.1016/j.jnutbio.2025.110019 40617306

[15] Yin M Li S Liu M Zhu W Chen Y Qiu W GUCY1A1-LDHA Axis suppresses ferroptosis in cardiac ischemia-reperfusion injury. Circ Res. (2025) 137(7):986–1005. 10.1161/CIRCRESAHA.124.326029 40856046

[16] Liu Y Yang G Huo S Wu J Ren P Cao Y Lutein suppresses ferroptosis of cardiac microvascular endothelial cells via positive regulation of IRF in cardiac hypertrophy. Eur J Pharmacol. (2023) 959:176081. 10.1016/j.ejphar.2023.176081 37797674

[17] Shi P Song C Qi H Ren J Ren P Wu J Up-regulation of IRF3 is required for docosahexaenoic acid suppressing ferroptosis of cardiac microvascular endothelial cells in cardiac hypertrophy rat. J Nutr Biochem. (2022) 104:108972. 10.1016/j.jnutbio.2022.108972 35227883

[18] Zhang Z Tang J Song J Xie M Liu Y Dong Z Elabela alleviates ferroptosis, myocardial remodeling, fibrosis and heart dysfunction in hypertensive mice by modulating the IL-6/STAT3/GPX4 signaling. Free Radical Biol Med. (2022) 181:130–42. 10.1016/j.freeradbiomed.2022.01.020 35122997

[19] Stockwell BR Friedmann Angeli JP Bayir H Bush AI Conrad M Dixon SJ Ferroptosis: a regulated cell death nexus linking metabolism, redox biology, and disease. Cell. (2017) 171(2):273–85. 10.1016/j.cell.2017.09.021 28985560 PMC5685180

[20] Zhao F Satyanarayana G Zhang Z Zhao J Ma X-L Wang Y. Endothelial autophagy in coronary microvascular dysfunction and cardiovascular disease. Cells. (2022) 11(13):2081. 10.3390/cells11132081 35805165 PMC9265562

[21] Zhang X Zhou H Chang X. Involvement of mitochondrial dynamics and mitophagy in diabetic endothelial dysfunction and cardiac microvascular injury. Arch Toxicol. (2023) 97(12):3023–35. 10.1007/s00204-023-03599-w 37707623

[22] Dixon SJ Lemberg KM Lamprecht MR Skouta R Zaitsev EM Gleason CE Ferroptosis: an iron-dependent form of nonapoptotic cell death. Cell. (2012) 149(5):1060–72. 10.1016/j.cell.2012.03.042 22632970 PMC3367386

[23] Feng Y Madungwe NB Imam Aliagan AD Tombo N Bopassa JC. Liproxstatin-1 protects the mouse myocardium against ischemia-reperfusion injury by decreasing VDAC1 levels and restoring GPX4 levels. Biochem Biophys Res Commun. (2019) 520(3):606–11. 10.1016/j.bbrc.2019.10.006 31623831 PMC7457545

[24] Chen B Fan P Song X Duan M. The role and possible mechanism of the ferroptosis-related SLC7A11/GSH/GPX4 pathway in myocardial ischemia-reperfusion injury. BMC Cardiovasc Disord. (2024) 24(1):531. 10.1186/s12872-024-04220-3 39354361 PMC11445876

[25] Zheng J Conrad M. Ferroptosis: when metabolism meets cell death. Physiol Rev. (2025) 105(2):651–706. 10.1152/physrev.00031.2024 39661331

[26] Flórez AF Alborzinia H. Ferroptosis: concepts and definitions. Adv Exp Med Biol. (2021) 1301:1–5. 10.1007/978-3-030-62026-4_1 34370284

[27] Čepelak I Dodig S Dodig DČ. Ferroptosis: regulated cell death. Arh Hig Rad Toksikol. (2020) 71(2):99–109. 10.2478/aiht-2020-71-3366 PMC7968485 32975106

[28] Galluzzi L Vitale I Aaronson SA Abrams JM Adam D Agostinis P Molecular mechanisms of cell death: recommendations of the Nomenclature committee on cell death 2018. Cell Death Differ. (2018) 25(3):486–541. 10.1038/s41418-017-0012-4 29362479 PMC5864239

[29] Shi J Gao W Shao F. Pyroptosis: gasdermin-mediated programmed necrotic cell death. Trends Biochem Sci. (2017) 42(4):245–54. 10.1016/j.tibs.2016.10.004 27932073

[30] Li J- Liu S- Yao R- Tian Y- Yao Y-. A novel insight into the fate of cardiomyocytes in ischemia-reperfusion injury: from iron metabolism to ferroptosis. Front Cell Dev Biol. (2021) 9:799499. 10.3389/fcell.2021.799499 34926476 PMC8675329

[31] Ye J Lyu T-J Li L-Y Liu Y Zhang H Wang X Ginsenoside Re attenuates myocardial ischemia-reperfusion induced ferroptosis via miR-144-3p/SLC7A11. Phytomedicine. (2023) 113:154681. 10.1016/j.phymed.2023.154681 36893674

[32] Wang Y Zhang Y Li J Zhao R Long X Li C Role of Mydgf in the regulation of hypoxia/reoxygenation-induced apoptosis in cardiac microvascular endothelial cells. In Vitro Cell Dev Biol Anim. (2022) 58(8):669–78. 10.1007/s11626-022-00709-3 36006589

[33] Diao L Wu Y Jiang X Chen B Zhang W Chen L Human umbilical cord mesenchymal stem cell-derived exosomes modulate the NLRP3 inflammasome/caspase-1 pathway to repress pyroptosis induced by hypoxia/reoxygenation in cardiac microvascular endothelial cells. Int Heart J. (2024) 65(6):1107–17. 10.1536/ihj.23-500 39477491

[34] Wu X Zhao X Li F Wang Y Ou Y Zhang H MLKL-mediated endothelial necroptosis drives vascular damage and mortality in systemic inflammatory response syndrome. Cell Mol Immunol. (2024) 21(11):1309–21. 10.1038/s41423-024-01217-y 39349742 PMC11527879

[35] Xiang Q Yi X Zhu X-H Wei X Jiang D-S. Regulated cell death in myocardial ischemia-reperfusion injury. Trends Endocrinol Metab. (2024) 35(3):219–34. 10.1016/j.tem.2023.10.010 37981501

[36] Du B Fu Q Yang Q Yang Y Li R Yang X Different types of cell death and their interactions in myocardial ischemia-reperfusion injury. Cell Death Discov. (2025) 11(1):87. 10.1038/s41420-025-02372-5 40044643 PMC11883039

[37] Monvoisin A Alva JA Hofmann JJ Zovein AC Lane TF Iruela-Arispe ML. VE-cadherin-CreERT2 transgenic mouse: a model for inducible recombination in the endothelium. Dev Dyn. (2006) 235(12):3413–22. 10.1002/dvdy.20982 17072878

[38] Payne S De Val S Neal A. Endothelial-Specific cre mouse models. Arterioscler, Thromb, Vasc Biol. (2018) 38(11):2550–61. 10.1161/ATVBAHA.118.309669 30354251 PMC6218004

[39] Doll S Proneth B Tyurina YY Panzilius E Kobayashi S Ingold I ACSL4 dictates ferroptosis sensitivity by shaping cellular lipid composition. Nat Chem Biol. (2017) 13(1):91–8. 10.1038/nchembio.2239 27842070 PMC5610546

[40] Kagan VE Mao G Qu F Angeli JPF Doll S Croix CS Oxidized arachidonic and adrenic PEs navigate cells to ferroptosis. Nat Chem Biol. (2017) 13(1):81–90. 10.1038/nchembio.2238 27842066 PMC5506843

[41] Jin B Zhang Z Zhang Y Yang M Wang C Xu J Ferroptosis and myocardial ischemia-reperfusion: mechanistic insights and new therapeutic perspectives. Front Pharmacol. (2024) 15:1482986. 10.3389/fphar.2024.1482986 39411064 PMC11473306

[42] Qiu M Yan W Liu M. YAP Facilitates NEDD4L-mediated ubiquitination and degradation of ACSL4 to alleviate ferroptosis in myocardial ischemia-reperfusion injury. Can J Cardiol. (2023) 39(11):1712–27. 10.1016/j.cjca.2023.07.030 37541340

[43] Shuai T Lu Y Zhang Y Li M Chen L Fang P RNF5-mediated Ubiquitination of ACSL4 attenuates ferroptosis and confers cardioprotection against myocardial ischemia-reperfusion injury. Biochem Pharmacol. (2026) 243(Pt 1):117524. 10.1016/j.bcp.2025.117524 41203033

[44] Hentze MW Muckenthaler MU Galy B Camaschella C. Two to tango: regulation of mammalian iron metabolism. Cell. (2010) 142(1):24–38. 10.1016/j.cell.2010.06.028 20603012

[45] Tang L-J Zhou Y-J Xiong X-M Li N-S Zhang J-J Luo X-J Ubiquitin-specific protease 7 promotes ferroptosis via activation of the p53/TfR1 pathway in the rat hearts after ischemia-reperfusion. Free Radical Biol Med. (2021) 162:339–52. 10.1016/j.freeradbiomed.2020.10.307 33157209

[46] Wang X Wang Y Li Z Qin J Wang P. Regulation of ferroptosis pathway by ubiquitination. Front Cell Dev Biol. (2021) 9:699304. 10.3389/fcell.2021.699304 34485285 PMC8414903

[47] Clemente LP Rabenau M Tang S Stanka J Cors E Stroh J Dynasore blocks ferroptosis through combined modulation of iron uptake and inhibition of mitochondrial respiration. Cells. (2020) 9(10):2259. 10.3390/cells9102259 33050207 PMC7650611

[48] Harrison PM Arosio P. The ferritins: molecular properties, iron storage function and cellular regulation. Biochim Biophys Acta. (1996) 1275(3):161–203. 10.1016/0005-2728(96)00022-9 8695634

[49] Mancias JD Wang X Gygi SP Harper JW Kimmelman AC. Quantitative proteomics identifies NCOA4 as the cargo receptor mediating ferritinophagy. Nature. (2014) 509(7498):105–9. 10.1038/nature13148 24695223 PMC4180099

[50] Hou W Xie Y Song X Sun X Lotze MT Zeh HJ Autophagy promotes ferroptosis by degradation of ferritin. Autophagy. (2016) 12(8):1425–8. 10.1080/15548627.2016.1187366 27245739 PMC4968231

[51] Kuno S Fujita H Tanaka Y- Ogra Y Iwai K. Iron-induced NCOA4 condensation regulates ferritin fate and iron homeostasis. EMBO Rep. (2022) 23(5):e54278. 10.15252/embr.202154278 35318808 PMC9066066

[52] Donovan A Lima CA Pinkus JL Pinkus GS Zon LI Robine S The iron exporter ferroportin/Slc40a1 is essential for iron homeostasis. Cell Metab. (2005) 1(3):191–200. 10.1016/j.cmet.2005.01.003 16054062

[53] Kruszewski M. Labile iron pool: the main determinant of cellular response to oxidative stress. Mutat Res. (2003) 531(1-2):81–92. 10.1016/j.mrfmmm.2003.08.004 14637247

[54] Li S Yang Y Li W. Human ferroportin mediates proton-coupled active transport of iron. Blood Adv. (2020) 4(19):4758–68. 10.1182/bloodadvances.2020001864 33007076 PMC7556139

[55] Ben Zichri-David S Shkuri L Ast T. Pulling back the mitochondria’s iron curtain. Npj Metab Health Dis. (2025) 3(1):6. 10.1038/s44324-024-00045-y 40052109 PMC11879881

[56] Mccarthy RC Kosman DJ. Ferroportin and exocytoplasmic ferroxidase activity are required for brain microvascular endothelial cell iron efflux. J Biol Chem. (2013) 288(24):17932–40. 10.1074/jbc.M113.455428 23640881 PMC3682590

[57] Nemeth E Tuttle MS Powelson J Vaughn MB Donovan A Ward DMV Hepcidin regulates cellular iron efflux by binding to ferroportin and inducing its internalization. Science. (2004) 306(5704):2090–3. 10.1126/science.1104742 15514116

[58] Taleb M Maillet I Le Bert M Mura C. Targeted autophagy disruption reveals the central role of macrophage iron metabolism in systemic iron homeostasis. Blood. (2022) 140(4):374–87. 10.1182/blood.2021014493 35472080

[59] Jiang L Wang J Wang K Wang H Wu Q Yang C RNF217 Regulates iron homeostasis through its E3 ubiquitin ligase activity by modulating ferroportin degradation. Blood. (2021) 138(8):689–705. 10.1182/blood.2020008986 33895792 PMC8394904

[60] Ding Y Hong T. Low doses of ozone alleviate cardiomyocyte ferroptosis induced by hypoxia-reoxygenation injury via the AMPK-mTOR pathway. Eur J Med Res. (2025) 30(1):558. 10.1186/s40001-025-02829-4 40604957 PMC12217297

[61] Zheng H Ou J Han H Lu Q Shen Y. SS-31@Fer-1 alleviates ferroptosis in hypoxia/reoxygenation cardiomyocytes via mitochondrial targeting. Biomed Pharmacother. (2025) 183:117832. 10.1016/j.biopha.2025.117832 39848110

[62] Han Y Yuan H Li F Yuan Y Zheng X Zhang X Ammidin ameliorates myocardial hypoxia/reoxygenation injury by inhibiting the ACSL4/AMPK/mTOR-mediated ferroptosis pathway. BMC Complement Med Ther. (2023) 23(1):459. 10.1186/s12906-023-04289-x 38102654 PMC10722690

[63] Ishimaru K Ikeda M Miyamoto HD Furusawa S Abe K Watanabe M Deferasirox targeting ferroptosis synergistically ameliorates myocardial ischemia reperfusion injury in conjunction with cyclosporine A. J Am Heart Assoc. (2024) 13(1):e031219. 10.1161/JAHA.123.031219 38158218 PMC10863836

[64] Ding Y Li W Peng S Zhou G Chen S Wei Y Puerarin protects against myocardial ischemia-reperfusion injury by inhibiting ferroptosis. Biol Pharm Bull. (2023) 46(4):524–32. 10.1248/bpb.b22-00174 36696989

[65] Yao D Bao L Wang S Tan M Xu Y Wu T Isoliquiritigenin alleviates myocardial ischemia-reperfusion injury by regulating the Nrf2/HO-1/SLC7a11/GPX4 axis in mice. Free Radical Biol Med. (2024) 221:1–12. 10.1016/j.freeradbiomed.2024.05.012 38734270

[66] GaoY HuangF ZhangY LaiD ZhaoJ LuY Multi-omics analysis of early reperfused ischemic heart reveals ERR*β*/*γ* activation protects against acute myocardial infarction injury. J Adv Res. (2025) S2090-1232(25):01011–2. 10.1016/j.jare.2025.12.02141429340

[67] Simonson B Chaffin M Hill MC Atwa O Guedira Y Bhasin H Single-nucleus RNA sequencing in ischemic cardiomyopathy reveals common transcriptional profile underlying end-stage heart failure. Cell Rep. (2023) 42(2):112086. 10.1016/j.celrep.2023.112086 36790929 PMC10423750

[68] Rochette L Dogon G Rigal E Zeller M Cottin Y Vergely C. Lipid peroxidation and iron metabolism: two corner stones in the homeostasis control of ferroptosis. Int J Mol Sci. (2022) 24(1):449. 10.3390/ijms24010449 36613888 PMC9820499

[69] Ursini F Maiorino M. Lipid peroxidation and ferroptosis: the role of GSH and GPx4. Free Radical Biol Med. (2020) 152:175–85. 10.1016/j.freeradbiomed.2020.02.027 32165281

[70] Han F Li S Yang Y Bai Z. Interleukin-6 promotes ferroptosis in bronchial epithelial cells by inducing reactive oxygen species-dependent lipid peroxidation and disrupting iron homeostasis. Bioengineered. (2021) 12(1):5279–88. 10.1080/21655979.2021.1964158 34402724 PMC8806540

[71] Nemeth E Rivera S Gabayan V Keller C Taudorf S Pedersen BK IL-6 mediates hypoferremia of inflammation by inducing the synthesis of the iron regulatory hormone hepcidin. J Clin Invest. (2004) 113(9):1271–6. 10.1172/JCI20945 15124018 PMC398432

[72] Wrighting DM Andrews NC. Interleukin-6 induces hepcidin expression through STAT3. Blood. (2006) 108(9):3204–9. 10.1182/blood-2006-06-027631 16835372 PMC1895528

[73] Ganz T Nemeth E. Hepcidin and iron homeostasis. Biochim Biophys Acta. (2012) 1823(9):1434–43. 10.1016/j.bbamcr.2012.01.014 22306005 PMC4048856

[74] Ajioka RS Phillips JD Kushner JP. Biosynthesis of heme in mammals. Biochim Biophys Acta. (2006) 1763(7):723–36. 10.1016/j.bbamcr.2006.05.005 16839620

[75] Duan G Li J Duan Y Zheng C Guo Q Li F Mitochondrial iron metabolism: the crucial actors in diseases. Molecules. (2022) 28(1):29. 10.3390/molecules28010029 36615225 PMC9822237

[76] Chen Y Guo X Zeng Y Mo X Hong S He H Oxidative stress induces mitochondrial iron overload and ferroptotic cell death. Sci Rep. (2023) 13(1):15515. 10.1038/s41598-023-42760-4 37726294 PMC10509277

[77] Li J Jia Y- Ding Y- Bai J Cao F Li F. The crosstalk between ferroptosis and mitochondrial dynamic regulatory networks. Int J Biol Sci. (2023) 19(9):2756–71. 10.7150/ijbs.83348 37324946 PMC10266069

[78] Fang J Yuan Q Du Z Fei M Zhang Q Yang L Ferroptosis in brain microvascular endothelial cells mediates blood-brain barrier disruption after traumatic brain injury. Biochem Biophys Res Commun. (2022) 619:34–41. 10.1016/j.bbrc.2022.06.040 35728282

[79] Zhang H Kim H Park BW Noh M Kim Y Park J CU06-1004 Enhances vascular integrity and improves cardiac remodeling by suppressing edema and inflammation in myocardial ischemia-reperfusion injury. Exp Mol Med. (2022) 54(1):23–34. 10.1038/s12276-021-00720-w 34997212 PMC8814060

[80] Li W Zhao X Zhang R Liu X Qi Z Zhang Y Ferroptosis inhibition protects vascular endothelial cells and maintains integrity of the blood-spinal cord barrier after spinal cord injury. Neural Regen Res. (2023) 18(11):2474–81. 10.4103/1673-5374.371377 37282479 PMC10360107

[81] Guo X-W Zhang H Huang J-Q Wang S-N Lu Y Cheng B PIEZO1 Ion channel mediates ionizing radiation-induced pulmonary endothelial cell ferroptosis via ca²⁺/calpain/VE-cadherin signaling. Front Mol Biosci. (2021) 8:725274. 10.3389/fmolb.2021.725274 34568428 PMC8458942

[82] Zhang H Zhou S Sun M Hua M Liu Z Mu G Ferroptosis of endothelial cells in vascular diseases. Nutrients. (2022) 14(21):4506. 10.3390/nu14214506 36364768 PMC9656460

[83] Conway D Schwartz MA. Lessons from the endothelial junctional mechanosensory complex. F1000 Biol Rep. (2012) 4:1. 10.3410/B4-1 22238515 PMC3251317

[84] Wang X Liu P Han L Tuo Y Chen H Wang X Suppressive effects of lovastatin on OGD/hemin-induced inflammation and ferroptosis in brain microvascular endothelial cells through the METTL3/HDAC6 cascade. Shock. (2025). 10.1097/SHK.0000000000002688. PMID: 40961409 40961409

[85] He X Tian K Lin X Chen X Su Y Lu Z Unveiling the role of RhoA and ferroptosis in vascular permeability: implications for osteoarthritis. Int J Mol Med. (2024) 54(4):86. 10.3892/ijmm.2024.5410 39129277 PMC11335351

[86] Kim JW Nam SA Koh E-S Kim HW Kim S Woo JJ The impairment of endothelial autophagy accelerates renal senescence by ferroptosis and NLRP3 inflammasome signaling pathways with the disruption of endothelial barrier. Antioxidants. (2024) 13(8):886. 10.3390/antiox13080886 39199133 PMC11351978

[87] janaszak-Jasiecka A Płoska A Wierońska JM Dobrucki LW Kalinowski L. Endothelial dysfunction due to eNOS uncoupling: molecular mechanisms as potential therapeutic targets. Cell Mol Biol Lett. (2023) 28(1):21. 10.1186/s11658-023-00423-2 36890458 PMC9996905

[88] Karbakhsh Ravari F Ghasemi Gorji M Rafiei A. From iron-driven cell death to clot formation: the emerging role of ferroptosis in thrombogenesis. Biomed Pharmacother. (2025) 189:118328. 10.1016/j.biopha.2025.118328 40628161

[89] Ziegler M Wang X Peter K. Platelets in cardiac ischaemia/reperfusion injury: a promising therapeutic target. Cardiovasc Res. (2019) 115(7):1178–88. 10.1093/cvr/cvz070 30906948 PMC6529900

[90] Wang B Wang Y Zhang J Hu C Jiang J Li Y ROS-induced lipid peroxidation modulates cell death outcome: mechanisms behind apoptosis, autophagy, and ferroptosis. Arch Toxicol. (2023) 97(6):1439–51. 10.1007/s00204-023-03476-6 37127681

[91] Ao Q Hu H Huang Y. Ferroptosis and endoplasmic reticulum stress in rheumatoid arthritis. Front Immunol. (2024) 15:1438803. 10.3389/fimmu.2024.1438803 39076977 PMC11284608

[92] Tang J Zhu J Xie H Song L Xu G Li W Mitochondria-Specific molecular crosstalk between ferroptosis and apoptosis revealed by *in situ* Raman spectroscopy. Nano Lett. (2024) 24(7):2384–91. 10.1021/acs.nanolett.3c05039 38341873

[93] Lee Y-S Kalimuthu K Park YS Luo X Choudry MHA Bartlett DL BAX-dependent mitochondrial pathway mediates the crosstalk between ferroptosis and apoptosis. Apoptosis. (2020) 25(9-10):625–31. 10.1007/s10495-020-01627-z 32737652 PMC7529973

[94] Jiang L Kon N Li T Wang S-J Su T Hibshoosh H Ferroptosis as a p53-mediated activity during tumour suppression. Nature. (2015) 520(7545):57–62. 10.1038/nature14344 25799988 PMC4455927

[95] Zheng Y Huang Y Xu Y Sang L Liu X Li Y. Ferroptosis, pyroptosis and necroptosis in acute respiratory distress syndrome. Cell Death Discov. (2023) 9(1):91. 10.1038/s41420-023-01369-2 36898986 PMC10000361

[96] Khawas S Sharma N. Cell death crosstalk in respiratory diseases: unveiling the relationship between pyroptosis and ferroptosis in asthma and COPD. Mol Cell Biochem. (2025) 480(3):1305–26. 10.1007/s11010-024-05062-5 39112808

[97] Ramos S Hartenian E Santos JC Walch P Broz P. NINJ1 Induces plasma membrane rupture and release of damage-associated molecular pattern molecules during ferroptosis. EMBO J. (2024) 43(7):1164–86. 10.1038/s44318-024-00055-y 38396301 PMC10987646

[98] Wu Y-T Zhang G-Y Hua Y Fan H-J Han X Xu H-L Ferrostatin-1 suppresses cardiomyocyte ferroptosis after myocardial infarction by activating Nrf2 signaling. J Pharm Pharmacol. (2023) 75(11):1467–77. 10.1093/jpp/rgad080 37738327

[99] Tu H Zhou Y-J Tang L-J Xiong X-M Zhang X-J Ali Sheikh MS Combination of ponatinib with deferoxamine synergistically mitigates ischemic heart injury via simultaneous prevention of necroptosis and ferroptosis. Eur J Pharmacol. (2021) 898:173999. 10.1016/j.ejphar.2021.173999 33675785

[100] Scarpellini C Klejborowska G Lanthier C Hassannia B Vanden Berghe T Augustyns K. Beyond ferrostatin-1: a comprehensive review of ferroptosis inhibitors. Trends Pharmacol Sci. (2023) 44(12):902–16. 10.1016/j.tips.2023.08.012 37770317

[101] Stairley RA Trouten AM Li S Roddy PL Deleon-Pennell KY Lee K-H Anti-Ferroptotic treatment deteriorates myocardial infarction by inhibiting angiogenesis and altering immune response. Antioxidants. (2024) 13(7):769. 10.3390/antiox13070769 39061839 PMC11273385

[102] Chen R Zhang Y Zhang H Zhou H Tong W Wu Y SGLT2 Inhibitor dapagliflozin alleviates intramyocardial hemorrhage and adverse ventricular remodeling via suppressing hepcidin in myocardial ischemia-reperfusion injury. Eur J Pharmacol. (2023) 950:175729. 10.1016/j.ejphar.2023.175729 37100110

[103] Zhang CH Yan YJ Luo Q. The molecular mechanisms and potential drug targets of ferroptosis in myocardial ischemia-reperfusion injury. Life Sci. (2024) 340:122439. 10.1016/j.lfs.2024.122439 38278348

[104] Li M Wu J Yang T Zhao Y Ren P Chang L Engineered biomimetic nanoparticles-mediated targeting delivery of allicin against myocardial ischemia-reperfusion injury by inhibiting ferroptosis. Int J Nanomed. (2024) 19:11275–92. 10.2147/IJN.S478276 PMC11550785 39524923

[105] Liao H Zhang X Lu W Sun Y Shi S Lin Y. Protection of framework nucleic acid complexes via regulating ferroptosis on myocardial ischemia-reperfusion injury. ACS Appl Mater interfaces. (2025) 17(23):33664–77. 10.1021/acsami.5c06755 40424601

